# Cholinergic Basal Forebrain Volumes Predict Gait Decline in Parkinson's Disease

**DOI:** 10.1002/mds.28453

**Published:** 2020-12-31

**Authors:** Joanna Wilson, Alison J. Yarnall, Chesney E. Craig, Brook Galna, Sue Lord, Rosie Morris, Rachael A. Lawson, Lisa Alcock, Gordon W. Duncan, Tien K. Khoo, John T. O'Brien, David J. Burn, John‐Paul Taylor, Nicola J. Ray, Lynn Rochester

**Affiliations:** ^1^ Translational and Clinical Research Institute Newcastle University Newcastle upon Tyne United Kingdom; ^2^ The Newcastle upon Tyne NHS Foundation Trust Newcastle upon Tyne United Kingdom; ^3^ Health, Psychology and Communities Research Centre, Department of Psychology Manchester Metropolitan University Manchester United Kingdom; ^4^ School of Biomedical, Nutritional and Sport Sciences Newcastle University Newcastle upon Tyne United Kingdom; ^5^ Auckland University of Technology Auckland New Zealand; ^6^ Department of Sport, Exercise, and Rehabilitation Northumbria University Newcastle upon Tyne United Kingdom; ^7^ Centre for Clinical Brain Sciences University of Edinburgh Edinburgh United Kingdom; ^8^ NHS Lothian Edinburgh United Kingdom; ^9^ School of Medicine & Menzies Health Institute Queensland Griffith University Gold Coast Queensland Australia; ^10^ School of Medicine, University of Wollongong Australia; ^11^ Department of Psychiatry University of Cambridge Cambridge United Kingdom; ^12^ Population Health Sciences Institute Newcastle University Newcastle upon Tyne United Kingdom

**Keywords:** Parkinson's disease; structural MRI; gait; NBM; acetylcholine

## Abstract

**Background:**

Gait disturbance is an early, disabling feature of Parkinson's disease (PD) that is typically refractory to dopaminergic medication. The cortical cholinergic system, originating in the nucleus basalis of Meynert of the basal forebrain, has been implicated. However, it is not known if degeneration in this region relates to a worsening of disease‐specific gait impairment.

**Objective:**

To evaluate associations between sub‐regional cholinergic basal forebrain volumes and longitudinal progression of gait impairment in PD.

**Methods:**

99 PD participants and 47 control participants completed gait assessments via an instrumented walkway during 2 minutes of continuous walking, at baseline and for up to 3 years, from which 16 spatiotemporal characteristics were derived. Sub‐regional cholinergic basal forebrain volumes were measured at baseline via MRI and a regional map derived from post‐mortem histology. Univariate analyses evaluated cross‐sectional associations between sub‐regional volumes and gait. Linear mixed‐effects models assessed whether volumes predicted longitudinal gait changes.

**Results:**

There were no cross‐sectional, age‐independent relationships between sub‐regional volumes and gait. However, nucleus basalis of Meynert volumes predicted longitudinal gait changes unique to PD. Specifically, smaller nucleus basalis of Meynert volume predicted increasing step time variability (*P* = 0.019) and shortening swing time (*P* = 0.015); smaller posterior nucleus portions predicted shortening step length (*P* = 0.007) and increasing step time variability (*P* = 0.041).

**Conclusions:**

This is the first study to demonstrate that degeneration of the cortical cholinergic system predicts longitudinal progression of gait impairments in PD. Measures of this degeneration may therefore provide a novel biomarker for identifying future mobility loss and falls. © 2020 The Authors. *Movement Disorders* published by Wiley Periodicals LLC on behalf of International Parkinson and Movement Disorder Society.

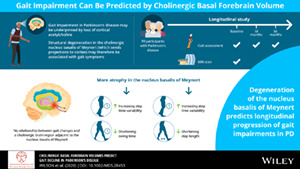

## Graphical abstract

March Infographic: Cholinergic Basal Forebrain Volumes Predict Gait Decline in Parkinson's Disease

Gait impairment is a common, debilitating feature of Parkinson's disease (PD) that manifests in early and even prodromal disease stages.^1^, ^2^, ^3^ It impacts quality of life^4^ and is associated with increased falls risk,^5^ cognitive decline,^6^ and reduced activity.^7^ Gait can be described through quantitatively measured characteristics^8^ reflecting selective gait alterations in response to ageing and disease.^9^, ^10^

Dopaminergic medications can immediately improve some aspects of PD gait,^11^, ^12^, ^13^ yet some characteristics continue to worsen over time despite optimal dopaminergic treatment.^10^ Current understanding of the neuroanatomical and functional substrates underpinning gait impairment is poor;^14^ establishing the non‐dopaminergic neurotransmitter systems involved in gait would enable development of intervention strategies that more effectively target gait decline in early PD.

Emerging evidence suggests the cholinergic system is involved in PD gait control.^15^, ^16^ Reduced thalamic cholinergic innervation, originating from the pedunculopontine nucleus‐laterodorsal tegmental complex (PPN), has been associated with falls,^17^ freezing of gait,^18^ and balance disturbance^19^ in PD. Furthermore, reduced PPN structural integrity has been associated with postural instability and gait disturbance in primates^20^ and PD.^21^

Cortical cholinergic denervation may also relate to PD gait impairment.^15^ Slower walking speed and shorter step length have been linked to reduced short‐latency afferent inhibition (SAI),^22^ indicative of less cortical cholinergic activity. Slower walking speed has also been associated with cortical cholinergic denervation and not thalamic cholinergic denervation.^23^, ^24^, ^25^ The nucleus basalis of Meynert (NBM), within the cholinergic basal forebrain (cBF), provides the major cholinergic input to the cortex. This region has been notably linked to cognitive changes seen in age and age‐related diseases.^26^, ^27^, ^28^, ^29^ Taken with the well‐established relationship between discrete gait impairments and cognitive impairment in PD,^6^, ^30^ this implies that NBM degeneration may be implicated in gait impairments. Indeed, recent work has identified a cross‐sectional association between NBM integrity and fast walking speed ~10 years after PD diagnosis.^31^

Although cross‐sectional studies provide insights, longitudinal studies are needed to understand the neural mechanisms contributing to gait decline and to identify biomarkers to monitor and predict these changes with progressing disease, within a timeframe where interventions would be most beneficial. We have shown that cerebrospinal fluid (CSF) β‐amyloid 1–42 levels, potentially linked to cortical cholinergic neurotransmission,^32^ predict gait decline in PD.^10^ As cBF Lewy body aggregates and neuronal losses occur early in disease,^17^ cBF volumetric measures may provide early biomarkers of gait decline. Recent work has identified that NBM volumes quantified in very early disease can predict future cognitive decline in PD.^26^, ^28^ However, whether these volumetric measures can also predict gait decline has not been investigated.

This study aimed to investigate the cortical cholinergic underpinnings of PD gait impairment. Using stereotactic mapping of the cBF, specific aims were to; (1) explore the relationship of sub‐regional cBF volumes with gait, and (2) assess the ability of sub‐regional cBF volumes to predict disease‐specific progression of gait impairments over 3 years. We hypothesized that NBM volumes, rather than an adjacent cBF region that projects to the hippocampus, would most strongly relate to PD gait. Additionally, characteristics related to gait pace and variability, which are strongly linked with cognition,^30^ were predicted to most strongly relate to NBM volume.

## Methods

### Participants

Participants with idiopathic PD and age‐matched controls were recruited to the Incidence of Cognitive Impairment in Cohorts with Longitudinal Evaluation‐PD study (ICICLE‐PD), with optional additional recruitment into the collaborative ICICLE‐GAIT study. Recruitment was conducted between June 2009 and December 2011.^33^, ^34^ Exclusion criteria included cognitive impairment (Mini‐Mental State Examination [MMSE] ≤24), diagnosis of parkinsonian disorders other than PD and poor command of the English language. Clinical and gait assessments were completed at three sessions: baseline, 18 months, and 36 months. MRI was completed at baseline. Idiopathic PD was diagnosed according to the Queen Square Brain Bank criteria^35^; diagnoses were reviewed at each assessment to reduce misdiagnosis risk. Participants were tested “on” dopaminergic medication for all assessments (1 hour after medication).

Participants within the current analysis were those with baseline MRI and gait data available. Baseline assessments were a median of 5.1 months after diagnosis in this restricted cohort of 100 PD and 47 control participants. This time elapsed from diagnosis to baseline assessment is comparable to some previous studies of gait impairment in PD (mean of 5^36^ and 7 months^37^ from diagnosis in previous cohort studies), although the severity of motor symptoms (mean MDS‐UPDRS III) of our cohort was higher (25 compared to 16 and 21, respectively). Other cohorts have assessed PD participants with a longer disease duration.^38^, ^39^, ^40^ The mean age at baseline in this study (66.5 years) compares well with these other cohorts (mean baseline age of 62–68 years across studies).

One PD brain image was excluded after manual image inspection; therefore, 99 PD participants had data available for use. The study was approved by the Newcastle and North Tyneside Research and Ethics Committee.

### Clinical Assessments

Age, sex, height, mass, years of education, disease duration, acetylcholinesterase (AChE) inhibitor prescription, and depression (Geriatric Depression Scale [GDS‐15]) were recorded. The National Adult Reading Test (NART) assessed premorbid intelligence at baseline. Global cognition was assessed through the MMSE and Montreal Cognitive Assessment (MoCA). PD‐specific motor severity was assessed through the Movement Disorders Society Unified Parkinson's Disease Rating Scale part three (MDS‐UPDRS III), from which Hoehn & Yahr stage (H&Y) was derived. Levodopa (l‐dopa) equivalent daily dose (LEDD) was calculated.^41^

### Gait Assessments

Participants walked at their self‐selected pace for 2 minutes around a 25‐m oval circuit that included a 7‐m long instrumented walkway (Platinum model GAITRite, CIR Systems, Franklin, NJ). At least 40 steps were completed over the walkway per participant to ensure robust measurement of gait variability.^42^ Gait outcomes were derived and quantified according to an a priori model developed for older adults and validated in PD,^8^ that describes 16 discrete gait characteristics within five domains. Methods to calculate the gait variables have been described previously.^42^

### MRI Acquisition

MRI acquisition was performed using a 3T Philips Intera Achieva scanner (Philips Medical Systems, Eindhoven, The Netherlands). A magnetization‐prepared rapid acquisition gradient echo (MP‐RAGE) T_1_‐weighted sequence produced high‐resolution structural images with the following parameters: repetition time = 9.6 ms, echo time = 4.6 ms, flip angle = 8°, SENSE factor = 2, field of view = 240 × 240 mm, voxel size = 1.5 × 1.5 × 1.5 mm^3^, acquisition time = 4 minutes; 150 sagittal slices (slice thickness = 1.2 mm) were taken.

### cBF Stereotactic Map

Stereotactic mapping of cBF nuclei was used to create the cBF map, as described by Kilimann et al.^43^ Briefly, the map was derived from a brain specimen of a 56‐year‐old male who died from myocardial infarction. This brain specimen underwent histological preparation and post‐mortem MRI scans, both in situ and after the brain was dehydrated for histological preparation. Mesulam's nomenclature^44^ was followed to identify cholinergic nuclei on digital pictures of stained brain slices, these were manually transferred into corresponding MR slices of the dehydrated brain. The MRI scan of the dehydrated brain was transformed into the space of the post‐mortem in situ scan, using an initial 12‐parameter affine transformation followed by a high‐dimensional nonlinear registration between the two scans.^45^ This was transferred to Montreal Neurological Institute (MNI) standard space to enable use of the high‐dimensional DARTEL (diffeomorphic anatomic registration using exponentiated lie algebra) registration method.^46^ The final stereotactic map distinguishes different cBF subdivisions, including cholinergic cell clusters corresponding to the medial septum, vertical and horizontal limb of the diagonal band, and the NBM.

### Image Pre‐Processing

T_1_‐weighted MRI scans were automatically segmented into gray matter (GM), white matter, and CSF partitions using the segmentation routine of the VBM8 toolbox (http://dbm.neuro.uni-jena.de/wordpress/vbm/download/) running under SPM12 (http://www.fil.ion.ucl.ac.uk/spm/software/spm12/). Each participant's resultant gray and white matter partitions were registered to MNI space using DARTEL.^46^ GM segments were warped using individual flow fields resulting from DARTEL registration. Voxel values were modulated for volumetric changes introduced by high‐dimensional normalization, enabling for total GM volume before warping to be preserved. Pre‐processed GM maps were visually inspected by one of three authors (J.W., C.E.C., and/or N.J.R.) for segmentation and registration accuracy. One PD participant did not pass inspection and their data was removed.

**FIG. 1:**
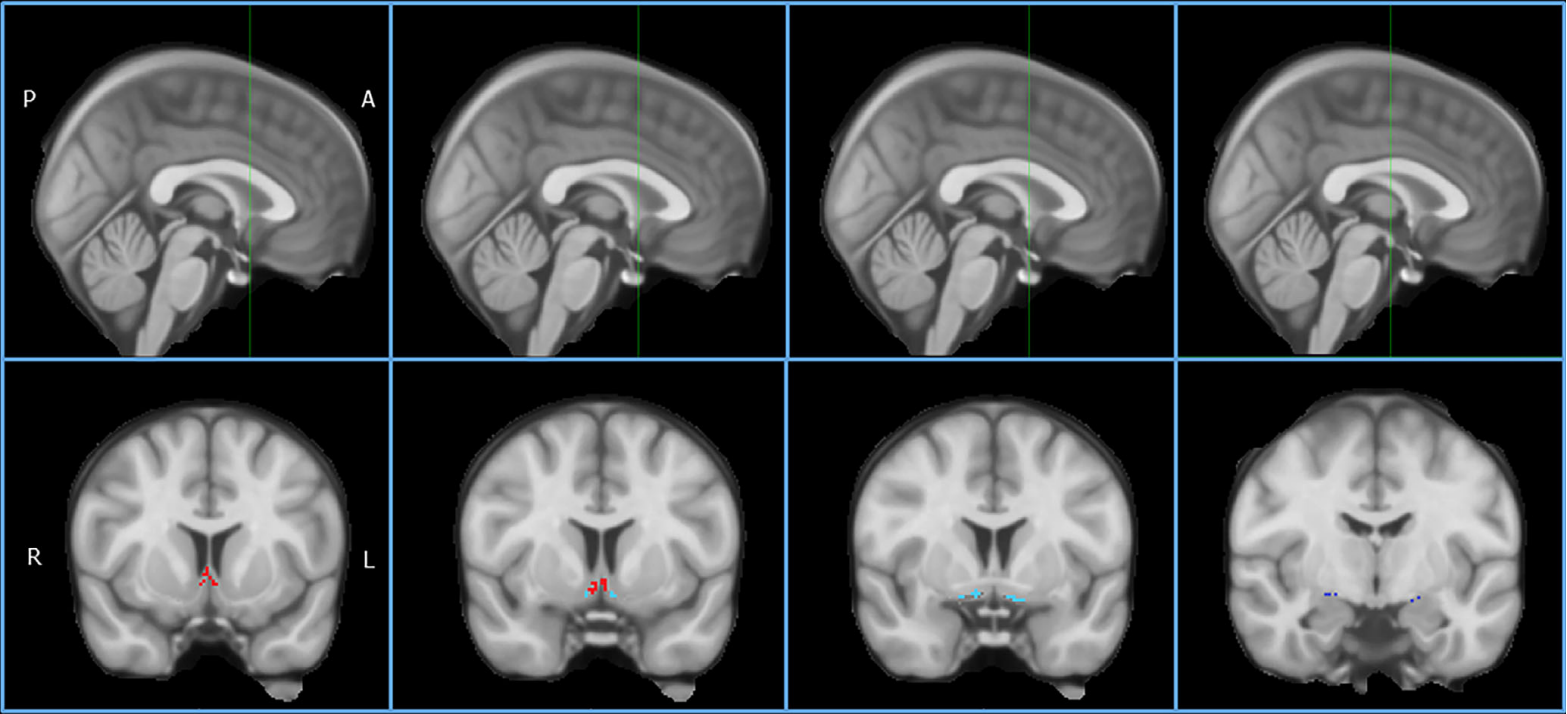
Cholinergic basal forebrain regions of interest. (Slices from left to right are coronal slices 9, 6, 3, and −8, as demonstrated in the top row. In the bottom row, the Ch1‐2 region of interest is demarcated in the first and second images, corresponding to the medial septum and vertical limb of the diagonal band; the Ch4 region of interest, corresponding to the NBM, is highlighted in the second and third images; the Ch4p region of interest, corresponding to the posterior NBM, is shown in the fourth image.) [Color figure can be viewed at wileyonlinelibrary.com]

Region‐specific cBF GM volumes were calculated as means of the total modulated GM voxel values within respective region‐of‐interest masks in template space. These volumes and global GM were normalized using regression by total intracranial volume (TIV, sum of total GM, white matter, and CSF volumes as a proxy for head size). Following previous PD literature using this cBF mask,^26^ regions‐of‐interest selected for analysis were: (1) a combination of the medial septum (Ch1) and vertical limb of the diagonal band (Ch2), (2) the NBM (Ch4), and (3) a posterior NBM subdivision (Ch4p) (Fig. 1). TIV‐normalized global GM volumes were additionally extracted and included as confounders in analyses to confirm that associations with cBF volumes were not explained by overall GM atrophy.

### Statistical Analysis

Analyses were conducted using SPSS (IBM Corp. V.24, USA) and R software (R Foundation for Statistical Computing, V3.5.2, Austria). The distribution of continuous variables was tested for normality through Kolmogorov–Smirnov tests and boxplot and histogram inspections.

#### Clinical Comparisons

Student's *t*‐tests, Mann–Whitney *U* tests, or χ^2^ tests examined baseline clinical characteristic differences between control and PD participants and, within the PD group, between those that did (“completers”) and did not (“non‐completers”) complete gait assessment at 36 months. Paired *t*‐tests or Wilcoxon signed‐rank tests assessed differences in baseline and final assessments of clinical characteristics within diagnostic groups.

#### Baseline Gait and cBF Volumes

One‐way analysis of covariance (ANCOVA), age and sex corrected, separately assessed differences in baseline gait and sub‐regional cBF volumes between control and PD participants, and between PD completers and non‐completers. Pearson's correlation and Pearson's partial correlation, age and sex controlled, tested within‐group associations between baseline gait and cBF volumes. Because of substantial skewness, gait asymmetry data were square root‐transformed and temporal variability data log transformed for cross‐sectional analyses.

#### Gait Change Over 3 Years

Linear mixed‐effects models (LMEM; R, “lme4”^47^ and “lmerTest”^48^) separately modeled change in each gait characteristic over 3 years. Although some participants withdrew or were lost to follow up across the 36‐month study period, all participants with at least two gait assessments were considered in longitudinal analyses; LMEM can appropriately estimate regression coefficients despite drop‐out of this nature.^49^ This approach can effectively handle the hierarchical nature of longitudinal, repeated‐measures data, without the need to remove participants with missing data. Random slope models gave each participant a unique intercept and slope, allowing for correlation between intercept and slope. Baseline age and sex were included as fixed effects, and model fit was assessed by likelihood ratio tests.

Changes in each gait characteristic over time were separately modeled within each group. Next, in the full sample, the interaction between group and time was additionally modeled to assess group differences in gait change. The effects of changes in l‐dopa medication over time on gait change were assessed through inspection of the interaction between LEDD and time.

#### cBF Volumes as Predictors of Gait Change

Gait characteristics identified as significantly changing over time in PD, and where change was different from change in controls, were selected for further analysis. LMEM determined the predictive utility of baseline cBF volumes for future gait changes in PD participants. Specifically, the interaction between cBF volumes and time was modeled with adjustment for baseline age, sex, and TIV‐normalized whole brain GM volumes as fixed effects. Sub‐regional volumes were included in separate models.

#### Multiple Comparisons

Group comparisons of clinical and gait characteristics, and significant changes in gait, were considered significant at *P* < 0.05, uncorrected. This allowed us to restrict subsequent analyses to gait characteristics, with exploratory‐level evidence, that are affected by PD. The NBM (Ch4) has previously been implicated in PD degeneration^26^, results for this region were considered significant at *P* < 0.05. Posterior NBM (Ch4p), the medial septum, and vertical limb of the diagonal band (Ch1‐2) regions‐of‐interest were Benjamini‐Hochberg corrected for multiple comparisons across the two regions in PD. There were no region‐specific predictions in control participants, therefore Benjamini‐Hochberg corrections were applied across the three cBF sub‐regions. Non‐corrected *P* values are reported.

## Results

### Participants

A total of 127 PD and 93 control participants consented to both studies (Supplementary Figure S1). After exclusions, 99 PD and 47 control participants had baseline imaging and gait data available. At 18 months, 89 (90%) PD participants and 37 (79%) control participants completed gait assessment; at 36 months, 69 (70%) PD and 35 (74%) control participants attended. A total of 67 (68%) PD and 33 (70%) control participants attended all assessments; 91 (92%) PD and 39 (83%) control participants attended at least two gait assessments and were therefore considered within longitudinal evaluations. The mean time to baseline assessment from PD diagnosis was 6 months and from the first subjective motor symptom was 27 months.

### Group Comparisons

At baseline, PD and control groups were age and sex matched, with no significant differences in height, mass, years of education, or NART score (Table 1). PD participants had greater cognitive impairment and depression scores (MMSE; *U* = 1673, *P* = 0.004: MoCA; *U* = 1234, *P* < 0.001: GDS‐15; *U* = 1099, *P* < 0.001). A total of 23% of PD participants were H&Y stage I at baseline, 59% were H&Y stage II, and 18% were H&Y stage III at baseline. At baseline, 89 (90%) PD participants were on dopaminergic medication; no participants were on AChE inhibitors. A total of 12 of 16 gait characteristics demonstrated greater impairment in PD than controls (Supplementary Figure S2). There were no differences between PD and control groups in any sub‐regional cBF volume (posterior NBM [Ch4p]: F_1,142_ = 1.17, *P* = 0.282; NBM [Ch4]: F_1,142_ = 3.35, *P* = 0.069; medial septum and vertical limb of the diagonal band [Ch1‐2]: F_1,142_ = 0.88, *P* = 0.351). PD completers were not significantly different from non‐completers for any clinical, gait, or imaging measure at baseline (Supplementary Table S1, Appendix S1).

**TABLE 1:** Clinical gait and cBF characteristics of participants at baseline

Characteristic	Parkinson's disease	Controls	Statistic, *P* value
Clinical assessments			
N	99	47	–
Age, years	66.53 (10.69)	65.82 (8.02)	*t* = −0.45, *P* = 0.683
Sex, males^a^	66 (67)	29 (62)	χ^2^ = 0.35, *P* = 0.557
Height, m	1.70 (0.08)	1.71 (0.10)	*t* = 0.87, *P* = 0.386
Mass, kg	78.93 (15.12)	79.53 (13.05)	*t* = 0.23, *P* = 0.817
Education, years	13.4 (4.0)	13.8 (3.7)	*U* = 2197, *P* = 0.584
NART	115.7 (10.3)	117.7 (7.9)	*U* = 2152, *P* = 0.586
MoCA	25.2 (3.6)	27.7 (2.0)	*U* = 1234, *P* < 0.001*
MMSE	28.7 (1.3)	29.3 (0.9)	*U* = 1673, *P* = 0.004*
GDS‐15	2.6 (2.2)	0.9 (1.2)	*U* = 1099, *P* < 0.001*
MDS‐UPDRS III	25.2 (10.2)	–	–
H&Y Stage^a^	23 I (23), 58 II (59), 18 III (18)	–	–
Disease duration, months	6.5 (4.8)	–	–
LEDD (mg/day)	171 (129)	–	–
Gait assessments			
Step velocity, ms^−1^	1.13 (0.21)	1.29 (0.14)	**F_(1,142)_ = 24.3,** ***P*** < 0.001*
Step length, m	0.63 (0.10)	0.69 (0.07)	F_(1,142)_ = 19.6, *P* < 0.001*
Swing time var, ms	17.71 (6.03)	13.92 (3.52)	F_(1,142)_ = 16.9, *P* < 0.001*
Step time var, ms	19.04 (6.41)	14.97 (4.17)	F_(1,142)_ = 17.3, *P* < 0.001*
Stance time var, ms	23.26 (9.74)	17.48 (5.46)	F_(1,142)_ = 16.3, *P* < 0.001*
Step velocity var, ms^−1^	0.05 (0.02)	0.05 (0.01)	F_(1,142)_ = 2.3, *P* = 0.103
Step length var, m	0.02 (0.01)	0.02 (0.01)	F_(1,142)_ = 7.1, *P* = 0.008*
Step time, ms	563.98 (46.29)	537.82 (45.15)	F_(1,142)_ = 9.6, *P* = 0.002*
Swing time, ms	394.28 (32.95)	386.06 (33.80)	F_(1,142)_ = 2.0, *P* = 0.157
Stance time, ms	734.10 (77.78)	690.73 (62.26)	F_(1,142)_ = 10.9, *P* = 0.001*
Step time asy, ms	20.02 (20.28)	11.44 (9.05)	F_(1,142)_ = 7.6, *P* = 0.007*
Swing time asy, ms	16.06 (15.48)	8.47 (7.89)	F_(1,142)_ = 12.0, *P* = 0.001*
Stance time asy, ms	15.49 (14.95)	8.95 (8.37)	F_(1,142)_ = 9.45, *P* = 0.003*
Step length asy, m	0.02 (0.02)	0.02 (0.02)	F_(1,142)_ = 0.5, *P* = 0.487
Step width, m	0.09 (0.03)	0.09 (0.02)	F_(1,142)_ = 0.4, *P* = 0.847
Step width var, m	0.02 (0.01)	0.02 (0.01)	F_(1,142)_ = 7.7, *P* = 0.006*
cBF assessments			
TIV normalized Ch4p volume, mm^3^	−0.004 (0.066)	0.008 (0.055)	F_1,142_ = 1.17, *P* = 0.282
TIV normalized Ch4 volume, mm^3^	−0.005 (0.049)	0.010 (0.044)	F_1,142_ = 3.35, *P* = 0.069;
TIV normalized Ch1‐2 volume, mm^3^	−0.003 (0.056)	0.007 (0.052)	F_1,142_ = 0.88, *P* = 0.351

All figures are mean (standard deviation) except ^a^ where figures are n (%). At baseline, n = 6 did not complete MoCA and n = 1 did not complete NART in the Parkinson's disease group. Significant differences are in bold, denoted by *.

Abbreviations: GDS‐15, Geriatric Depression Scale; NART, National Adult Reading Test; MoCA, Montreal Cognitive Assessment; MMSE, Mini‐Mental State Examination; MDS‐UPDRS III, Movement Disorders Society Unified Parkinson's disease rating scale part three; H&Y, Hoehn and Yahr; LEDD, Levodopa equivalent daily dose; var, variability (standard deviation); asy, asymmetry; cBF, cholinergic basal forebrain; TIV, total intracranial volume (note cBF volumes are TIV‐normalized using analysis of covariance).

### Baseline Associations Between Gait and cBF Volumes

In PD only, greater baseline step velocity and step length were correlated bivariately with larger volumes of all cBF sub‐regions (step velocity: |r| > 0.20, *P* < 0.04 in all sub‐regions; step length: |r| > 0.23, *P* < 0.02 in all sub‐regions, Supplementary Table S2, Appendix S1). After adjusting for age and sex within partial correlations, associations were no longer significant (|r| < 0.1, *P* > 0.05 in all sub‐regions, Supplementary Table S3, Appendix S1).

### Longitudinal Changes in Clinical and Gait Characteristics

Over 3 years, PD motor severity and LEDD increased (*P* < 0.001, Supplementary Table S4, Appendix S1). MMSE, but not MoCA, score worsened over three years in PD only (*P* = 0.034); depression did not significantly change in either group. Two PD participants were taking AChE inhibitors at 18 months and three were at 36 months.

Table 2 presents modeled change in each gait characteristic for PD and control participants, and the group–time interactions for each characteristic. Over 36 months, 5 of 16 characteristics declined significantly in PD; change was significantly different from change in controls for four of these (Supplementary Figure S3). Step length shortened by 8 mm per year, *P* = 0.001; step time variability increased by 0.9 ms per year, *P* = 0.026; step length variability increased by 1.4 mm per year, *P* < 0.001; and swing time shortened by 2.8 ms per year, *P* = 0.003. Although step time changed significantly over 36 months in PD (*P* = 0.011), change was not statistically different from controls (*P* = 0.228). As previously identified in the ICICLE cohort,^10^ change in LEDD over time was not associated with longitudinal gait change for any gait characteristic except step width variability (data not shown). LEDD has, therefore, not been included in any subsequent analyses involving gait change.

**TABLE 2:** Modeled changes in gait over 36 months in Parkinson's disease and controls, and group × time interactions for change

	Parkinson's disease				Controls				Group × time interaction			
	Δ per year	SE	*t*	*P*‐value	Δ per year	SE	*t*	*P*‐value	β	SE	*t*	*P*‐value
Step velocity (m/s)	−0.0068	0.0057	−1.21	0.231	0.0037	0.0061	0.61	0.549	−0.0102	0.0089	−1.14	0.257
Step length (m)	−0.0077	0.0023	−3.36	0.001*	0.0003	0.0019	0.16	0.878	−0.0079	0.0034	−2.31	0.024*
Swing time var (ms)	0.6193	0.3819	1.62	0.108	−0.3330	0.1859	−1.79	0.077	0.9321	0.5420	1.72	0.088
Step time var (ms)	0.8789	0.3849	2.28	0.026*	−0.3277	0.2432	−1.35	0.182	1.097	0.5434	2.02	0.046*
Stance time var (ms)	0.9556	0.5418	1.76	0.081	−0.4189	0.3025	−1.39	0.170	1.305	0.7710	1.69	0.093
Step velocity var (m/s)	0.0012	0.0007	1.60	0.114	−0.0004	0.0006	−0.66	0.509	0.0016	0.0011	1.48	0.142
Step length var (m)	0.0014	0.0004	3.56	<0.001*	0.0002	0.0002	0.67	0.507	0.0013	0.0006	2.22	0.029*
Step time (ms)	−3.128	1.210	−2.58	0.011*	−0.814	1.311	−0.62	0.539	−2.320	1.908	−1.22	0.228
Swing time (ms)	−2.821	0.927	−3.05	0.003*	1.462	0.836	1.75	0.091	−4.249	1.415	−3.00	0.003*
Stance time (ms)	−3.357	1.838	−1.83	0.072	−3.057	1.980	−1.54	0.134	−0.345	2.885	−0.12	0.905
Step time asy (ms)	0.3323	0.7680	0.43	0.666	−0.1487	0.4361	−0.34	0.734	0.456	1.097	0.42	0.678
Swing time asy (ms)	−0.6627	0.5495	−1.21	0.232	−0.3995	0.4265	−0.94	0.352	−0.3398	0.8063	−0.42	0.674
Stance time asy (ms)	−0.3704	0.6184	−0.60	0.552	−0.6038	0.4369	−1.38	0.172	0.0785	0.8607	0.09	0.927
Step length asy (m)	0.0011	0.0009	1.31	0.193	−0.0005	0.0006	−0.72	0.474	0.0015	0.0013	1.21	0.228
Step width (m)	0.0008	0.0007	1.14	0.259	−0.0002	0.0008	−0.26	0.798	0.0010	0.0011	0.90	0.369
Step width var (m)	0.0005	0.0003	1.62	0.109	−0.0001	0.0002	−0.28	0.781	0.0006	0.0004	1.25	0.212

Significant associations (*P* < 0.05) are in bold and denoted by *.

Abbreviations: var, variability; asy, asymmetry; SE, standard error; CI, confidence interval.

### cBF Volumes as Predictors of Gait Decline

**FIG. 2:**
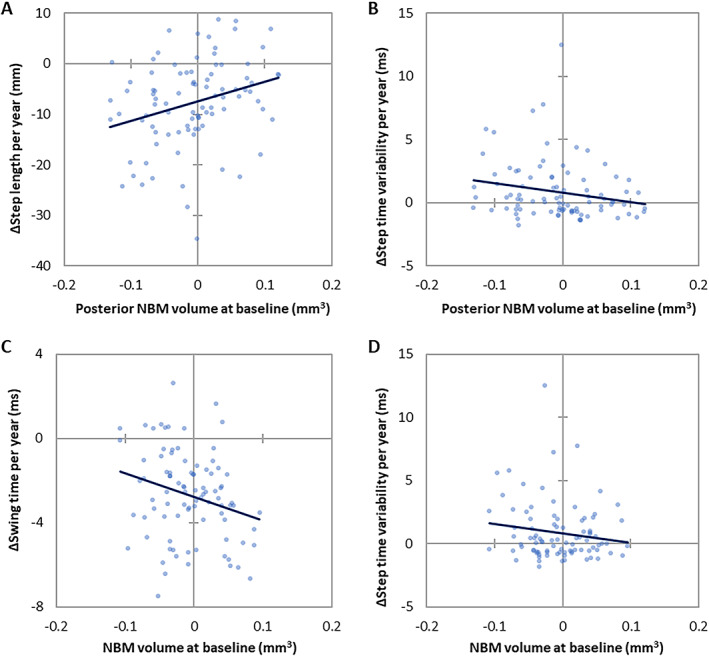
Correlation between sub‐regional cBF volumes at baseline and change in gait per year in Parkinson's disease. **A**: correlation between change in step length and baseline posterior NBM (Ch4p) volume. **B**: correlation between change in step time variability and baseline posterior NBM (Ch4p) volume. **C**: correlation between change in swing time and baseline NBM (Ch4) volume. **D**: correlation between change in step time variability and baseline NBM (Ch4) volume. Values for gait change per year were derived from the model coefficient for time, for individuals within the Parkinson's disease cohort.) [Color figure can be viewed at wileyonlinelibrary.com]

Table 3 summarizes the baseline cBF volume predictors of PD gait change. Smaller posterior NBM (Ch4p) volumes predicted greater step length shortening (β = 0.098, *P* = 0.007) and greater step time variability increase (β = −14.47, *P* = 0.019) over 36 months; the inclusion of posterior NBM volume into models improved model fit (χ^2^ = 7.77, *P* = 0.021; χ^2^ = 6.02, *P* = 0.049, respectively). Smaller NBM (Ch4) volumes also predicted greater step time variability increase (β = −17.20, *P* = 0.041), whereas larger NBM volumes predicted greater swing time shortening (β = −47.88, *P* = 0.015); NBM volume inclusion into models improved model fit (χ^2^ = 6.86, *P* = 0.032; χ^2^ = 6.85, *P* = 0.033, respectively). To illustrate findings, correlations between sub‐regional cBF volumes and change in gait per year for individuals, as derived from individual modeled trajectories of change, are plotted (Fig. 2).

**TABLE 3:** LMEM assessing sub‐regional cBF volumes as predictors of gait change in Parkinson's disease

Gait characteristic	Predictor region	Regression coefficients				
		β	SE	*t*	*P*‐value	CI
**Step length (m)**	**Ch4p**	0.0980	0.0353	2.78	**0.007** ^*^	(0.028, 0.169)
	**Ch4**	0.0806	0.0494	1.63	0.107	(−0.017, 0.181)
	**Ch1‐2**	0.0407	0.0421	0.97	0.336	(−0.042, 0.125)
**Step time variability (ms)**	**Ch4p**	−14.466	6.027	−2.40	**0.019** ^*^	(−26.6, −2.57)
	**Ch4**	−17.195	8.276	−2.08	**0.041** ^*^	(−34.0, −0.872)
	**Ch1‐2**	−11.376	6.979	−1.63	0.107	(−25.3, 2.42)
**Step length variability (m)**	**Ch4p**	−0.0043	0.0064	−0.67	0.506	(−0.017, 0.008)
	**Ch4**	0.0005	0.0087	0.06	0.955	(−0.017, 0.018)
	**Ch1‐2**	−0.0027	0.0073	−0.37	0.712	(−0.017, 0.012)
**Swing time (ms)**	**Ch4p**	−27.91	14.52	−1.92	0.058	(−56.5, 1.24)
	**Ch4**	−47.88	19.25	−2.49	**0.015** ^*^	(−85.8, −9.04)
	**Ch1‐2**	−16.08	16.96	−0.95	0.345	(−49.7, 18.6)

Baseline age, sex, and global gray matter volume were included as fixed effects in models. Significant predictors of changes in gait are in bold and denoted by *.

## Discussion

To our knowledge, this is the first study to assess the relationship between cBF volumes and PD gait dysfunction longitudinally. Assessments were completed in a relatively homogeneous PD cohort and well‐matched controls, enabling precise modeling of disease‐specific gait changes. Assessments were conducted at a time where intervention strategies may be most effective. We found no age‐independent cross‐sectional relationships between cBF volumes and gait. However, NBM volumes uniquely predicted future disease‐specific gait decline. In addition, gait pace and variability characteristics were most closely linked with NBM degeneration. These findings improve understanding of the cortical cholinergic underpinnings of gait impairment and imply that NBM volumetric measurement may serve as a predictive biomarker for PD gait changes.

### Cross‐Sectional Associations Are Mediated by Age

There was limited association between gait variables and sub‐regional cBF volumes in both PD and control groups at baseline, with only step velocity and step length identified in PD. However, this finding did not survive control for age and sex. Ray et al^26^ also found no cross‐sectional relationships between cBF volumes and cognitive scores after controlling for age in a separate cohort assessed after a similar disease duration. Speculatively, the effects of ageing on associations between the cBF and gait and cognition may outweigh the effects of PD in early disease, whereas ageing effects may become secondary to disease pathology as the disease progresses. This may explain the recent associations made between the NBM and fast walking speed in later stage PD.^31^ Repeating cross‐sectional analyses at a later disease point may give valuable insight to this suggestion.

As with previous reports,^26^, ^28^ we found no differences in cBF volumes in people with PD compared with controls. A lack of between‐group differences may be because of heterogeneity of cholinergic degeneration in early PD,^17^ with only a portion of early PD presumed to have cholinergic involvement. Additionally, volumetric changes may not be sensitive to very early structural changes in the region. Indeed, degeneration of white matter projections occurs before GM atrophy in PD,^50^ and NBM white matter tract integrity is more strongly associated with cognitive performance than NBM volume in healthy individuals.^51^

### NBM Volumes Predict Disease‐Specific Gait Decline

We wished to explore the predictive value of sub‐regional cBF volumes to understand the mechanisms of PD gait impairment, so restricted longitudinal analyses to the four gait characteristics that changed significantly and more greatly than in controls. These were step length, step time variability, step length variability, and swing time. Of these, only step length variability changes were not predicted by baseline NBM volumes. Analyses controlled for both TIV and total GM volume, giving us confidence in the precision of the identified associations between the NBM and gait. All assessments were completed “on” dopaminergic medication. Recent work has also identified an association between NBM integrity and fast walking speed as assessed “on”, but not “off,” dopaminergic medication.^31^ Speculatively, therefore, our findings may support the notion that the influence of the cholinergic system on gait is only evident when “confounding” effects of dopamine are accounted for through optimization of the dopaminergic system.

#### Step Time Variability

The relationship between baseline NBM volumes and step time variability change is consistent with data showing that CSF β‐amyloid 1–42 (linked to NBM atrophy in older adults^32^) is a predictor of step time variability change.^10^ Additionally, step time variability demonstrated disease‐specific, dopa‐resistant change in the entire ICICLE‐GAIT cohort over 3 years^10^ that was confirmed in the participants assessed here. This characteristic should therefore be considered a robust measure of gait change in PD, and the current data imply it is cholinergically mediated.

#### Step Length

Step length typically responds well to l‐dopa in early disease, because it closely reflects hypokinesia, whereas gait variability measures are not always responsive.^52^ The association between NBM volumes reported here and step length may indicate that step length is not purely dopaminergically controlled, supported by the previously identified association between step length and SAI.^22^ Step length has also been linked to the hippocampus in healthy older adults,^14^ which is thought to be involved in spatial navigation while walking.^53^ As such, it was surprising that no relationship between step length and volume of the hippocampal‐projecting Ch1‐2 region was identified.

#### Swing Time

Somewhat counterintuitively, larger baseline NBM volumes predicted greater reductions in swing time—typically considered a gait impairment—over 3 years. However, in PD, shortening swing time may reflect a compensatory strategy in which the timing of steps becomes shorter in an attempt to overcome shortening step length, thereby preserving overall walking speed.^54^ Shortening step timings as a compensatory strategy is thought to be controlled primarily by the cerebellum acting to increase primary motor cortex activity.^55^ Our findings may indicate that structurally preserved NBM volumes reflect a greater ability to use this compensatory mechanism, a suggestion supported by the known role of acetylcholine (ACh) in compensatory processes.^56^, ^57^

### The Role of Cognition

Cortical ACh is associated with cognition, specifically executive function and attention.^58^ These cognitive domains are strongly related to gait dysfunction in PD.^30^ Specifically, gait characteristics within the pace gait domain,^8^ including step length, have been linked to executive function and attention whereas global cognition has been linked to gait variability.^34^ Additionally, changes in fluctuating attention in early PD are predicted by step length, step length variability, and step time variability.^6^ The current study provides tentative evidence for a shared neural underpinning of gait and cognition that may originate within the NBM. Understanding the role of cognition in associations between gait and the cBF requires an in‐depth investigation of the effects of different cognitive domains on both gait and imaging parameters, as well as how these interact over time. The complexity of this warrants its own independent investigation and would form a useful follow‐on study.

### Clinical Implications

Primarily, this work aimed to further understanding of the cholinergic underpinnings of PD gait mechanistically. However, findings may also have clinical use. This study provides first evidence that cBF volumes can predict gait changes in PD. As such, these volumetric measures could be considered within a combinational battery of clinical biomarkers of gait progression, to monitor disease progression, and stratify patients in clinical trials. As gait changes and falls are closely related,^5^ whether NBM volumes could act as warning markers for those more likely to experience falls warrants further investigation.

This study has also provided further evidence for an association between the cortical cholinergic system and PD gait impairment, highlighting the need for novel therapies extending beyond the dopaminergic system. AChE inhibitors, which act to increase available ACh, can improve mobility measures in PD,^15^, ^52^ including gait speed and step time variability as a proxy marker for falls risk.^59^, ^60^ Overall, findings help strengthen the case for therapeutically targeting the cholinergic system to limit PD gait progression.

### Limitations

Some limitations should be noted. Attrition was notable at 30% over 3 years, comparable to similar studies.^61^ Mixed‐effects models allowed use of all data and reduced bias compared to traditional analytical approaches. Rates of changes in gait over time may have been underestimated, as non‐completers had greater disease severity at baseline than completers (although the difference between these was non‐significant). Assessments were completed in close succession (median of 3 weeks), yet gait and imaging assessments were dissociated in time and so correlation‐based analyses were required. Although the Ch4 region from the stereotactic atlas corresponds to cholinergic neurons within the NBM, the NBM has heterogeneous cell populations, and we cannot dismiss a contribution from these populations to findings. The stereotactic map was generated from one post‐mortem brain, suggesting that inter‐individual variability in volumes may not be optimally considered, although the efficient DARTEL algorithm normalized volumes in MNI space.^43^ This study aimed to further our understanding of the relationship between the cortical cholinergic system and PD gait impairment. Future investigations should also consider the involvement of subcortical cholinergic structures in PD gait, such as the PPN, to establish whether the cholinergic nuclei investigated here are uniquely associated with gait in PD, as has been previously found.^23^ Finally, as with all volumetric measures, cBF volumes may not directly reflect cholinergic activity, which must be assessed through molecular imaging targeting the cortical cholinergic system.^23^, ^24^ Multi‐modal neuroimaging approaches are required to fully understand the effect of cBF volume loss on cholinergic activity.

## Conclusions

In conclusion, NBM atrophy measured in PD can predict future disease‐specific gait changes. Findings reinforce the notion that gait control in PD involves the cortical cholinergic system, and that ACh should, therefore, be considered as a therapeutic target to mitigate gait dysfunction. Considered alongside the extant literature, NBM atrophy may underpin both motor and cognitive impairments. Further investigations of the interplay between NBM volume, gait and cognition must be considered to understand these complex relationships.

## Author Roles

(1) Research Project: A. Conception, B. Organization, C. Execution; (2) Statistical Analysis: A. Design, B. Execution, C. Review and Critique; (3) Manuscript Preparation: A. Writing the First Draft, B. Review and Critique.

J.W.: 2A, 2B, 3A, 3B

A.J.Y.: 1B, 1C, 3B

C.E.C.: 2B, 3B

B.G.: 1C, 2C, 3B

S.L.: 1B, 1C, 3B

R.M.: 1C, 3B

R.A.L.: 1C, 2C, 3B

L.A.: 1C, 3B

G.W.D.: 1B, 1C, 3B

T.K.K.: 1B, 1C, 3B

J.O.B.: 1A, 3B

D.B.: 1A, 3B

J‐P.T.: 2C, 3B

N.J.R.: 2B, 2C, 3B

L.R.: 1A, 1B, 1C, 2C, 3B

## Financial Disclosures

This work was funded by grants from Parkinson's UK (J‐0802, G‐1301) and Lockhart Parkinson's Disease. The work has been supported by the National Institute for Health Research (NIHR) Biomedical Research Unit based at Newcastle upon Tyne Hospitals NHS Foundation Trust and Newcastle University; the NIHR Newcastle BRC and Newcastle CRF Infrastructure funding. J.W. and N.J.R. are supported by the Wellcome Trust. A.Y. is supported by the Newcastle NIHR BRC plus grants from Parkinson's UK, Dunhill Medical Trust, Michael J. Fox Foundation, EU IMI, NIHR, and the Weston Brain Institute. She has received honoraria from Teva‐Lundbeck and sponsorship from Teva‐Lundbeck, UCB, GlaxoSmithKline, Genus, Britannia, and AbbVie for attending conferences. R.A.L is supported by grants from Parkinson's UK. J.O.B. is supported by the NIHR Cambridge Biomedical Research Centre, has acted as a consultant for Axon Neuroscience, TauRx, Eisai, GE Healthcare, and Lilly, and received grant support from Merck and Alliance Medical. D.J.B. is supported by grants from Parkinson's UK, National Institute for Health Research Health Technology Assessment (NIHR HTA), MRC, and Newcastle Healthcare Charity and has received consultancy from the Michael J. Fox Foundation and honorarium for lectures from Teva‐Lundbeck and Union Chimique Belge (UCB). J‐P.T. is supported by Newcastle NIHR BRC. He has received speaker fees from GE Healthcare, funding from Sosei‐Heptares and has consulted for Kyowa‐Kirin. L.R.'s research program is supported in part by grants from the Medical Research Council (MRC), European Union, Parkinson's UK, and the National Institute for Health Research Biomedical Research Unit (NIHR BRU) for Lewy Body Dementias.

## Supporting information


**FIG. S1**. Flowchart of participants recruited and assessed in ICICLE‐PD and ICICLE‐GAIT.

**FIG. S2**. Radar plot illustrating the pattern of gait impairment at baseline. (Var, variability; Asy, asymmetry. The central line represents control data. Deviations from zero along the axes radiating from the center of the plot represent how many standard deviations [z score based on control baseline means and standard deviations] the Parkinson's disease group differed from controls. Gait variables are organized by domain [Lord et al.].^8^ *Indicates significant differences between the control and Parkinson's disease groups.

**FIG. S3**. Distribution of gait characteristics at each assessment.

**APPENDIX S1** Supporting Information
