# Supplementary figures and images for: Cholinergic Basal Forebrain Volumes Predict Gait Decline in Parkinson's Disease
Source: Mov Disord. 2020 Dec 31;36(3):611–21. doi: 10.1002/mds.28453 (PMC8048433; doi:10.1002/mds.28453)

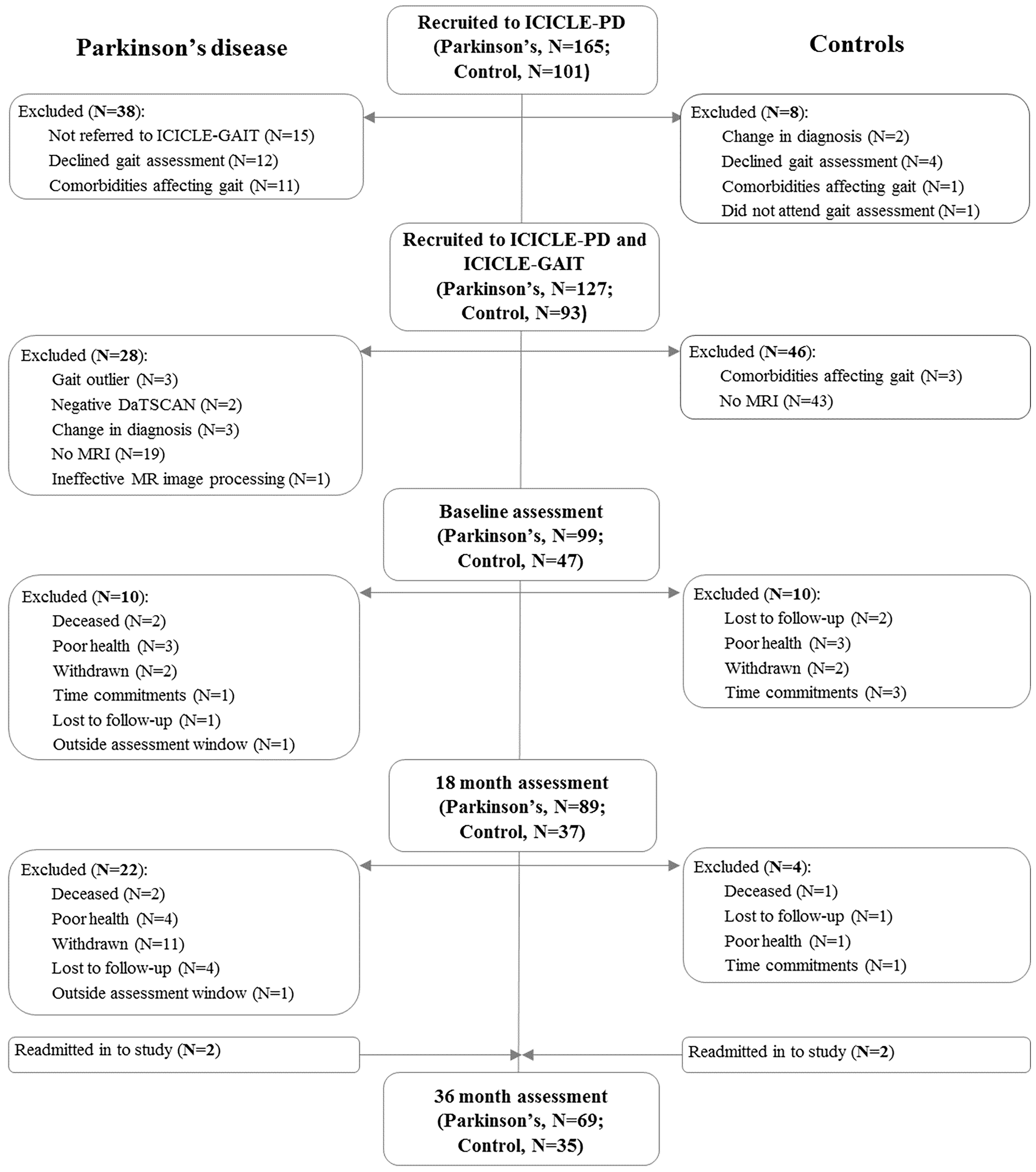

Supplement: Supplementary file 1 — FIG. S1. Flowchart of participants recruited and assessed in ICICLE‐PD and ICICLE‐GAIT. [file MDS-36-611-s002.tif]

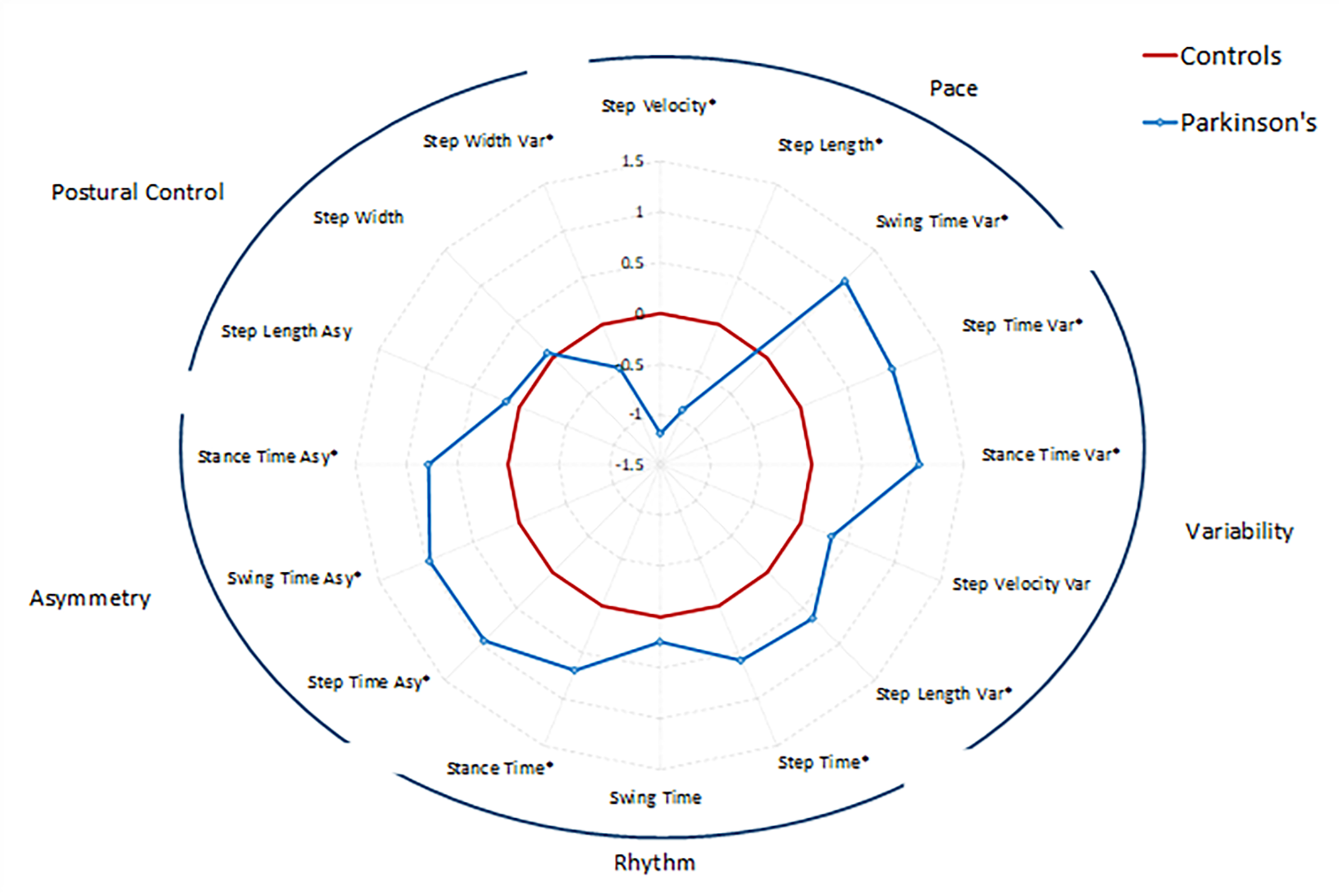

Supplement: Supplementary file 2 — FIG. S2. Radar plot illustrating the pattern of gait impairment at baseline. (Var, variability; Asy, asymmetry. The central line represents control data. Deviations from zero along the axes radiating from the center of the plot represent how many standard deviations [z score based on control baseline means and standard deviations] the Parkinson's disease group differed from controls. Gait variables are organized by domain [Lord et al.].8 *Indicates significant differences between the control and Parkinson's disease groups. [file MDS-36-611-s004.tif]

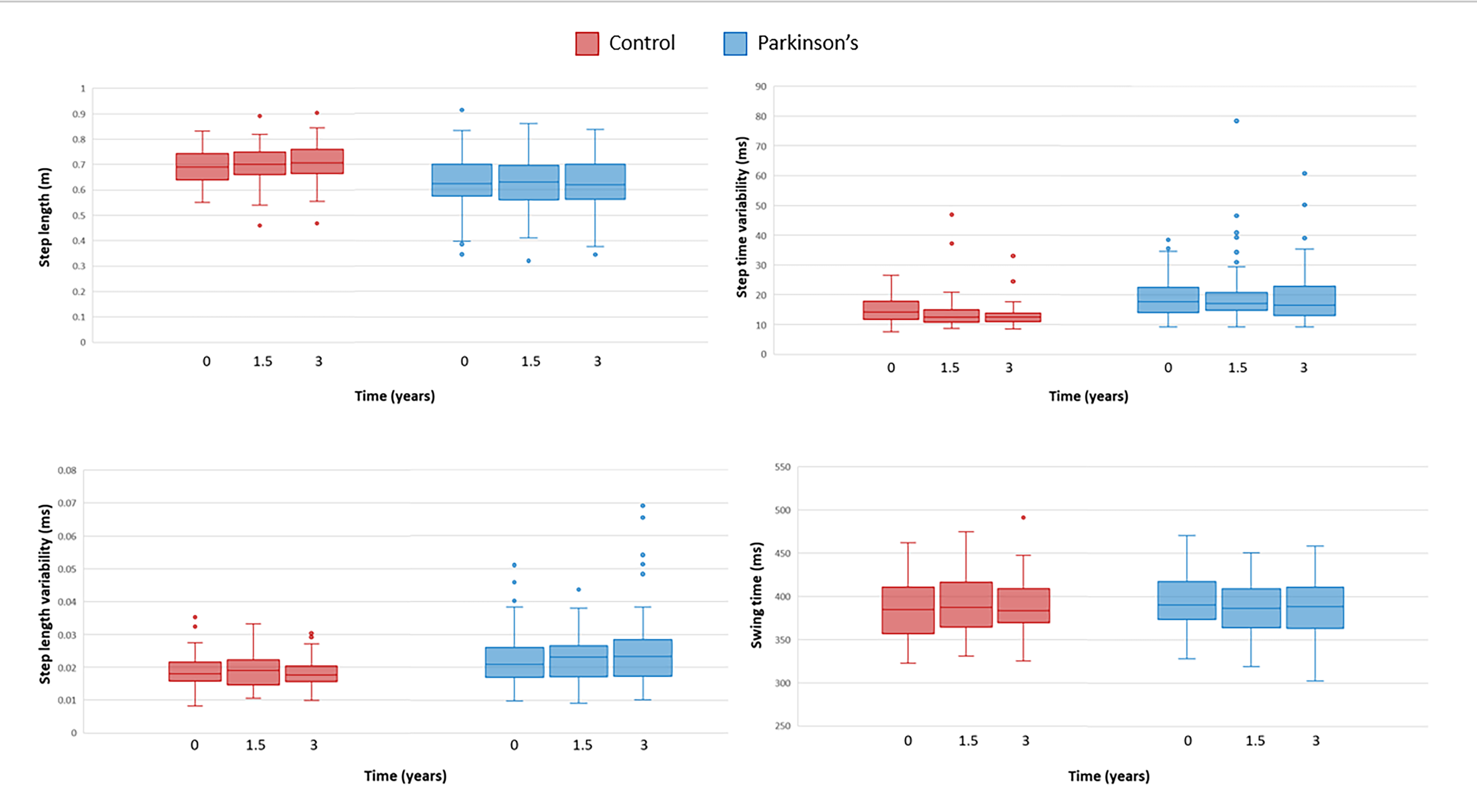

Supplement: Supplementary file 3 — FIG. S3. Distribution of gait characteristics at each assessment. [file MDS-36-611-s003.tif]
