# Supplementary material for: Cholinergic Basal Forebrain Volumes Predict Gait Decline in Parkinson's Disease
Source: Mov Disord. 2020 Dec 31;36(3):611–21. doi: 10.1002/mds.28453 (PMC8048433; doi:10.1002/mds.28453)
Supplement: Supplementary file 4 — APPENDIX S1 Supporting Information [file MDS-36-611-s001.docx]

**Supplementary Material**

**Supplementary table 1.** Demographic and clinical data for Parkinson’s disease participants who did and did not complete assessments at 36 months.

| **Characteristic** | **Completers (n=69)** | **Non-completers (n=30)** | **Group difference, p-value** |
| --- | --- | --- | --- |
| **Clinical assessments** | | | |
| Age, years | 65.36 (9.86) | 69.2 (12.0) | t=1.68, 0.095 |
| Sex, Males ⸣ | 45 (65) | 21 (70) | Χ^2^=0.07, 0.800 |
| Height, m | 1.70 (0.09) | 1.70 (0.07) | t=-0.30, 0.767 |
| Mass, kg | 78.04 (15.09) | 80.98 (15.25) | t=0.89 0.378 |
| Education, years | 13.7 (3.8) | 12.7 (4.5) | U=812, 0.239 |
| NART | 115.6 (10.7) | 116.0 (9.3) | U=975, 0.931 |
| MoCA | 25.6 (3.6) | 24.3 (3.7) | U=731, 0.123 |
| MMSE | 28.8 (1.1) | 28.4 (1.6) | U=918, 0.353 |
| GDS-15 | 2.6 (2.3) | 2.7 (1.9) | U=949, 0.507 |
| MDS-UPDRS III | 24.5 (10.2) | 26.8 (10.0) | t=1.02, 0.312 |
| H&Y Stage ⸣ | 19 I (27), 39 II (57), 11 III (16) | 4 I (13), 19 II (63), 7 III (23) | Χ^2^=1.77, 0.414 |
| Disease duration, months | 6.1 (4.1) | 7.5 (6.0) | U=930, 0.422 |
| LEDD | 152 (113) | 214 (154) | U=820, 0.098 |
| **Gait assessments** | | | |
| Step velocity, ms^-1^ | 1.15 (0.21) | 1.07 (0.23) | F_(1,95)_=1.4, p=0.245 |
| Step length, m | 0.64 (0.09) | 0.60 (0.11) | F_(1,95)_=1.1, p=0.300 |
| Swing time sd, ms | 16.86 (4.92) | 19.66 (7.77) | F_(1,95)_=2.0, p=0.159 |
| Step time sd, ms | 18.25 (5.60) | 20.86 (7.77) | F_(1,95)_=2.1, p=0.149 |
| Stance time sd, ms | 21.94 (8.73) | 26.30 (11.33) | F_(1,95)_=3.4, p=0.068 |
| Step velocity sd, ms^-1^ | 0.05 (0.01) | 0.06 (0.02) | F_(1,95)_=0.8, p=0.375 |
| Step length sd, m | 0.02 (0.01) | 0.02 (0.01) | F_(1,95)_=2.4, p=0.128 |
| Step time, ms | 561.36 (47.18) | 570.00 (54.19) | F_(1,95)_=0.7, p=0.405 |
| Swing time, ms | 394.81 (33.58) | 393.07 (31.99) | F_(1,95)_=0.01, p=0.917 |
| Stance time, ms | 728.27 (74.40) | 747.51 (84.84) | F_(1,95)_=1.1, p=0.304 |
| Step time asym, ms | 19.77 (19.05) | 20.61 (23.21) | F_(1,95)_=0.3, p=0.558 |
| Swing time asym, ms | 16.26 (15.09) | 15.60 (16.59) | F_(1,95)_=0.01, p=0.913 |
| Stance time asym, ms | 15.81 (14.88) | 14.74 (15.34) | F_(1,95)_=0.003, p=0.959 |
| Step length asym, m | 0.02 (0.02) | 0.03 (0.02) | F_(1,95)_=1.1, p=0.291 |
| Step width, m | 0.09 (0.03) | 0.10 (0.03) | F_(1,95)_=1.4, p=0.240 |
| Step width sd, m | 0.02 (0.01) | 0.02 (0.01) | F_(1,95)_=0.03, p=0.861 |
| **cBF assessments** | | | |
| TIV normalised Ch4p volume | 0.002 (0.062) | -0.018 (0.074) | F_1,95_ = 0.23, p=0.630 |
| TIV normalised Ch4 (NBM) volume | -0.001 (0.046) | -0.014 (0.056) | F_1,95_ = 0.06, p=0.813 |
| TIV normalised Ch1-2 volume | 0.002 (0.054) | -0.014 (0.059) | F_1,95_ = 0.26, p=0.614 |

[GDS-15, Geriatric Depression Scale; NART, National Adult Reading Test; MoCA, Montreal Cognitive Assessment; MMSE, Mini-Mental State Examination; MDS-UPDRS III, Movement Disorders Society Unified Parkinson’s disease Rating Scale part three; H&Y, Hoehn and Yahr; LEDD, Levodopa Equivalent Daily Dose; sd, standard deviation; asym, asymmetry; cBF, cholinergic basal forebrain [NB values are TIV normalised using ANCOVA]. All figures are mean (standard deviation) except ⸣ where figures are n(%). At baseline, n=6 did not complete MoCA and n=1 did not complete NART in the Parkinson’s group. Significant differences are in bold, denoted by *.]

**Supplementary table 2.** Bivariate Pearson correlations between gait characteristics and total intracranial volume normalised cBF volumes.

|  | **Parkinson’s Disease** | | | **Controls** | | |
| --- | --- | --- | --- | --- | --- | --- |
|  | **Ch4p** | **Ch4** | **Ch1-2** | **Ch4p** | **Ch4** | **Ch1-2** |
| **Step velocity** | ***.207 (.040)*** | ***.230 (.022)*** | ***.218 (.030)*** | .248 (.092) | .161 (.279) | .147 (.325) |
| **Step length** | ***.261 (.009)*** | ***.290 (.004)*** | ***.233 (.020)*** | .272 (.065) | .048 (.747) | .077 (.606) |
| **Swing time sd** | -.116 (.253) | -.081 (.423) | -.068 (.501) | -.058 (.700) | -.042 (.781) | -.133 (.374) |
| **Step time sd** | -.041 (.689) | .005 (.957) | .011 (.912) | -.236 (.111) | -.182 (.220) | -.191 (.198) |
| **Stance time sd** | -.034 (.741) | -.021 (.839) | -.049 (.633) | -.208 (.160) | -.154 (.302) | -.216 (.145) |
| **Step velocity sd** | -.004 (.970) | .027 (.788) | .016 (.876) | -.182 (.221) | -.084 (.572) | -.252 (.087) |
| **Step length sd** | -.147 (.148) | -.139 (.171) | -.072 (.478) | -.144 (.333) | -.041 (.784) | -.193 (.193) |
| **Step time** | .008 (.937) | .012 (.904) | -.064 (.528) | .028 (.850) | -.120 (.421) | -.095 (.527) |
| **Swing time** | .164 (.105) | .159 (.116) | .060 (.557) | .097 (.515) | -.047 (.755) | -.017 (.912) |
| **Stance time** | -.059 (.565) | -.050 (.620) | -.105 (.299) | -.010 (.948) | -.149 (.318) | -.126 (.397) |
| **Step time asy** | .113 (.266) | .061 (.550) | -.022 (.826) | -.007 (.965) | -.051 (.733) | -.172 (.248) |
| **Swing time asy** | .099 (.329) | .096 (.345) | .038 (.711) | -.011 (.941) | .075 (.615) | .073 (.624) |
| **Stance time asy** | .073 (.471) | .042 (.682) | -.012 (.909) | -.025 (.867) | .108 (.468) | .068 (.649) |
| **Step length asy** | -.040 (.691) | -.005 (.962) | .089 (.379) | -.116 (.436) | -.198 (.182) | -.155 (.297) |
| **Step width** | -.069 (.499) | -.018 (.857) | .060 (.555) | -.056 (.709) | -.050 (.738) | .123 (.409) |
| **Step width sd** | -.142 (.162) | -.034 (.741) | -.144 (.155) | -.125 (.403) | -.193 (.194) | -.019 (.898) |

[All values are presented as r (p). Significant correlations (under Benjamini-Hochberg correction) are highlighted and in bold.]

**Supplementary table 3.** Age and sex adjusted partial correlations between gait characteristics and total intracranial volume normalised cBF volumes.

|  | **Parkinson’s Disease** | | | **Controls** | | |
| --- | --- | --- | --- | --- | --- | --- |
|  | **Ch4p** | **Ch4** | **Ch1-2** | **Ch4p** | **Ch4** | **Ch1-2** |
| **Step velocity** | .018 (.858) | .058 (.572) | .067 (.514) | .181 (.235) | .094 (.539) | .110 (.474) |
| **Step length** | .009 (.931) | .058 (.573) | .020 (.846) | .163 (.284) | -.049 (.749) | -.011 (.941) |
| **Swing time sd** | .039 (.707) | .071 (.489) | .064 (.536) | -.001 (.993) | .024 (.876) | -.118 (.441) |
| **Step time sd** | .061 (.555) | .113 (.270) | .101 (.323) | -.176 (.246) | -.121 (.427) | -.162 (.289) |
| **Stance time sd** | .070 (.495) | .081 (.431) | .031 (.767) | -.127 (.407) | -.087 (.569) | -.178 (.243) |
| **Step velocity sd** | .044 (.668) | .082 (.423) | .058 (.573) | -.116 (.448) | -.050 (.746) | -.223 (.142) |
| **Step length sd** | .022 (.827) | .019 (.852) | .078 (.445) | -.017 (.910) | .098 (.522) | -.151 (.321) |
| **Step time** | -.020 (.846) | -.021 (.838) | -.108 (.295) | -.078 (.609) | -.180 (.238) | -.194 (.201) |
| **Swing time** | .022 (.832) | .017 (.869) | -.085 (.409) | -.094 (.539) | -.181 (.235) | -.144 (.346) |
| **Stance time** | -.036 (.725) | -.034 (.738) | -.101 (.323) | -.059 (.700) | -.159 (.298) | -.193 (.205) |
| **Step time asy** | -.064 (.534) | -.123 (.230) | -.195 (.056) | -.128 (.401) | -.131 (.392) | -.240 (.113) |
| **Swing time asy** | -.006 (.950) | -.005 (.962) | -.060 (.559) | .064 (.677) | .145 (.341) | .106 (.488) |
| **Stance time asy** | -.046 (.654) | -.078 (.446) | -.123 (.229) | -.017 (.914) | .126 (.411) | .077 (.614) |
| **Step length asy** | -.052 (.612) | -.014 (.894) | .108 (.294) | .017 (.911) | -.038 (.803) | -.149 (.330) |
| **Step width** | -.127 (.215) | -.064 (.536) | .042 (.686) | -.053 (.731) | -.003 (.982) | .126 (.410) |
| **Step width sd** | .039 (.701) | .166 (.105) | -.004 (.968) | -.013 (.930) | -.073 (.635) | .019 (.901) |

[All values are presented as r (p).]

**Supplementary table 4.** Clinical characteristics of participants at baseline and 36 months.

|  | **Parkinson’s disease** | | | **Control** | | |
| --- | --- | --- | --- | --- | --- | --- |
| **Characteristic** | **Baseline** | **36 months** | **Statistic, p-value** | **Baseline** | **36 months** | **Statistic, p-value** |
| **N** | 99 | 69 | - | 47 | 35 | - |
| **Age, years** | 66.53 (10.69) | 68.42 (9.86) | **t=-81.6, p<.001*** | 65.82 (8.02) | 70.20 (6.10) | **t=-83.3, p<.001*** |
| **Sex, Males ⸣** | 66 (67) | 45 (65) | - | 29 (62) | 21 (60) | - |
| **Height, m** | 1.70 (0.08) | 1.69 (0.09) | **t=3.60, p=.001*** | 1.71 (0.10) | 1.71 (0.09) | t=1.49, p=.145 |
| **Mass, kg** | 78.93 (15.12) | 79.05 (17.03) | t=-1.18, p=.244 | 79.53 (13.05) | 79.89 (13.00) | t=1.17, p=.252 |
| **Education, years** | 13.4 (4.0) | - | - | 13.8 (3.7) | - | - |
| **NART** | 115.7 (10.3) | - | - | 117.7 (7.9) | - | - |
| **MoCA** | 25.2 (3.6) | 26.3 (3.6) | W_63_=-1.50, p=.134 | 27.7 (2.0) | 28.3 (1.9) | W_35_=-1.20, p=.230 |
| **MMSE** | 28.7 (1.3) | 28.4 (2.0) | **W_69_=-2.12, p=.034*** | 29.3 (0.9) | 29.45(1.0) | W_35_=-0.19, p=.850 |
| **GDS-15** | 2.6 (2.2) | 2.5 (2.2) | W_69_=-0.59, p=.554 | 0.9 (1.2) | 0.8 (1.2) | W_35_=-0.87, p=.931 |
| **MDS-UPDRS III** | 25.2 (10.2) | 35.8 (11.8) | **W_69_=-6.14, p<.001*** | - | - | - |
| **H&Y Stage ⸣** | 23 I (23), 58 II (59), 18 III (18) | 1 I (1), 61 II (88), 7 III (10) | - | - | - | - |
| **Disease duration, months** | 6.5 (4.8) | - | - | - | - | - |
| **LEDD (mg/day)** | 171 (129) | 524 (258) | **W_69_=-7.06, p<.001*** | - | - | - |

- [GDS-15, Geriatric Depression Scale; NART, National Adult Reading Test; MoCA, Montreal Cognitive Assessment; MMSE, Mini-Mental State Examination; MDS-UPDRS III, Movement Disorders Society Unified Parkinson’s Disease Rating Scale; H&Y, Hoehn and Yahr; LEDD, Levodopa Equivalent Daily Dose. All figures are mean (standard deviation) except ⸣ where figures are n(%). At baseline, n=6 did not complete MoCA and n=1 did not complete NART in the Parkinson’s group. In controls, n=1 participant did not have height measured, nor completed MoCA or GDS-15 assessment at 36 months. Significant differences are in bold and denoted by *. NB, of the 18 H&Y stage III participants at baseline, 7 withdrew before assessment at 36 months and 4 “reverted” from stage II]
